# A study on the method and effect of the construction of a humanized mouse model of fecal microbiota transplantation

**DOI:** 10.3389/fmicb.2022.1031758

**Published:** 2022-11-16

**Authors:** Yaru Wang, Zhenzhen Zhang, Bin Liu, Chunzhi Zhang, Junying Zhao, Xianping Li, Lijun Chen

**Affiliations:** ^1^School of Biological Engineering, Dalian Polytechnic University, Liaoning, Dalian, China; ^2^National Engineering Research Center of Dairy Health for Maternal and Child, Beijing Sanyuan Foods Co. Ltd., Beijing, China; ^3^Beijing Engineering Research Center of Dairy, Beijing Technical Innovation Center of Human Milk Research, Beijing Sanyuan Foods Co. Ltd., Beijing, China

**Keywords:** fecal microbiota transplantation, humanization, gestation, specific pathogen-free mice, germ-free mice

## Abstract

The gestation period is critical for the health of the mother and fetus. Malnutrition or over nutrition during pregnancy may cause gestational diseases that can result in adverse pregnancy outcomes. Fecal microbiota transplantation (FMT) can be used to re-establish new gut microbiota to treat a variety of diseases and construct a model to investigate the nutritional health during pregnancy. Therefore, this study investigated whether human-derived gut microbiota during pregnancy could colonize the intestines of mice. Moreover, we determined the time and method of intervention for FMT. Based on this information, a humanized mouse model of FMT was constructed to simulate the human intestinal microecology during pregnancy, and serve as a useful animal model for the study of nutritional health and disease during pregnancy. Germ-free (GF) and specific pathogen free (SPF) C57BL/6J mice were selected for humanized gestational FMT and the transplantation outcomes were evaluated. The results demonstrated that the gestational intestinal microbiota colonized the intestines of mice, allowing researchers to construct a humanized mouse model of gestational FMT. The main intestinal flora of the gestational period were transplanted into GF mice, with the gestational flora being similar to the flora of GF mice after transplantation. However, antibiotics could not eliminate the original microbial flora in SPF mice, and the flora was complex and variable after FMT with little increase in abundance. Background flora had a significant impact on the outcomes assessment. The results were better in GF mice than in SPF mice, and after microbiota transplantation, a superior effect was observed on day 21 compared to days 7 and 14.

## Introduction

Approximately 10–100 trillion microorganisms are found closely associated with the human gastrointestinal tract (Cheng and Guo, 2012). The gastrointestinal tract and microbiota constitute an ecosystem that contributes to the integrity of the intestinal wall and maintains immune response (Zhang et al., 2019; Bartolini et al., 2020). The human microbiota is closely associated with the development and progression of many intestinal diseases (Giorgetti et al., 2015). Disruption of the balance of the gastrointestinal flora may have a significant impact on human health. Several studies have found a link between the gut microbiota and a variety of diseases during pregnancy, including gestational diabetes, inflammatory bowel disease, and gastrointestinal disorders (Giessen et al., 2019; Roth-schulze et al., 2021; Liu et al., 2022). Nutritional health during pregnancy is the foundation for the health and growth of infants and children (Jiang et al., 2018). Fecal microbiota transplantation (FMT) was first proposed in 1958 by Eiseman et al., who transplanted the functional flora from the feces of healthy individuals into the gastrointestinal tract of patients to re-establish a new intestinal microbiota for the treatment of intestinal and extraintestinal diseases (Eiseman et al., 1958). For decades, FMT has been considered a special type of organ transplantation and has been successfully used to eradicate pathogenic toxin-producing bacteria from patients with *Clostridium difficile* infection and for the treatment of various diseases, such as inflammatory bowel disease, ulcerative colitis, and irritable bowel syndrome (Zhang, 2013; Weingarden and Vaughn, 2017; Kumar and Fischer, 2019; Leonardi et al., 2020). Moreover, FMT provides new avenues for clinical treatment. Animal experiments on FMT have primarily been based on mouse models. Specific pathogen free (SPF) mice are inexpensive and easy to obtain, making them the preferred choice of many researchers. However, the presence of the original intestinal microbiota in SPF mice makes the colonization of new microbiota more difficult (Xu et al., 2021). Germ-free (GF) mice are free of bacteria, viruses, fungi, protozoa, and other putrefactive and parasitic organisms (Gordon and Pesti, 1971). Due to their uniqueness in lacking any microbiota, GF mice have an advantage over conventional laboratory animals for FMT (Tu et al., 2021). However, no uniform standards for mouse selection, outcome evaluation, as well as methods, or cycle of intervention for the humanized mouse model of FMT have been established, thus necessitating in-depth research in this regard. Therefore, in this study, humanized mouse models were constructed by transplanting fecal microbiota into SPF and GF mice to simulate human intestinal microecology during pregnancy. The intervention time, method, and transplantation outcomes of both mouse models were assessed to provide an experimental basis for the standardization of humanized mouse models for FMT in the future.

## Materials and methods

### Materials

#### Experimental animals

Forty eight-week-old GF-grade female C57BL/6J mice weighing 18–22 g were obtained form Jiangsu GemPharmatech Co., Ltd. (Production License No.: SYXK (Jiangsu) 2022-0004; Certificate No.: No.320727221100115413, No.32072722110010 1,478) and housed in Jiangsu GemPharmatech Co. Ltd. (Certificate No.: SYXK (Jiangsu) 2022-0004). The mice were acclimatized for 1 week with free access to water and food on a 12 h light/12 h dark cycle and were provided with the same irradiation genetically modified feed and sterile pure water purchased from Jiangsu Synergy Pharmaceutical Biology Co., Ltd. Room temperature was 23 ± 2°C and the relative humidity was 40–70%. This study was approved by the laboratory animal use and management committee of Jiangsu GemPharmatech Co., Ltd. (Welfare Ethics No.: GPTAP20220321-1).

Forty-eight-week-old SPF-grade female C57BL/6 J mice weighing 18–22 g were obtained from SPF (Beijing) Biotechnology Co., Ltd. (Production License No.: SCXK (Beijing) 2019-0010, certificate No.: 110324210104089445). The mice were housed in Sino Animal (Beijing) Science and Technology Development Co., Ltd. (License No.: SYXK 2020-0051). The mice were acclimatized for 1 week with free access to water and food on a 12 h light/12 h dark cycle. They were provided with the same irradiation- sterilized maintenance feed and sterile water purchased from SPF (Beijing) Biotechnology Co., Ltd. Room temperature was 20–26°C and the relative humidity was 40–70%. This study was approved by the Laboratory Animal Use and Management Committee of Sino Animal (Beijing) Science and Technology Development Co., Ltd. (Ethics No. 20210118YZE-3R).

#### Experimental reagents and equipment

Ampicillin (98%, Shanghai Macklin Biochemical Co., Ltd.), neomycin sulfate (United States Pharmacopoeia, (USP) grade, 680 I.U./mg, Shanghai Macklin Biochemical Co., Ltd.), metronidazole (99%, Shanghai Macklin Biochemical Co., Ltd.), vancomycin (USP, ≥950 μg/mg, Shanghai Macklin Biochemical Co., Ltd.), glycerol (Sangon Biotech (Shanghai) Co., Ltd.), biosafety cabinet (HR30/IIA2, Qingdao Haier Biomedical Co., Ltd.), electronic balance (BSA2202S, Sartorius AG), frozen centrifuge (X1R 75,004,250, Thermo Fisher Scientific, China), anaerobic incubator (BACTRON EZ/2, Shanghai Longyue Instrument Equipment Co., Ltd.), and cryogenic freezer (DW-86 L626, Haier Biomedical Co., Ltd.) were used.

### Experimental methods

#### Preparation of fecal microbiota solution

Stools were collected from nine healthy female volunteers at 30–40 weeks of gestation, and stools were collected from each volunteer for 15 consecutive days. All stool samples were collected between May and July 2021.The pregnant volunteers had not consumed antibiotics or any medication for 3 months and did not present with constipation, diarrhea, or other physical discomfort. The pooled stool samples (15 days) from nine pregnant volunteers were individually tested using metagenomic sequencing. A bar chart of the relative abundance of gut microbial composition at the genus level is provided (Supplementary Figure S1). According to the bar chart of the relative abundance of gut microbial composition at the genus level, the main genera in mixed feces of the nine pregnant women volunteers were *Bacteroides, Faecalibacterium, Roseburia*, *Eubacterium*.

After extraction, the fecal bacterial solution was stored at −80°C and expended in approximately 3 weeks. Previous studies have found that the extracted fecal fluids can be preserved for 2–12 months at −80°C (Hamilton et al., 2012; Ye and Wang, 2016; Lin et al., 2020). The feces from all nine donors were pooled; then, 160 mg of fecal samples was suspended in 1 ml of 0.9% normal saline and stored at −80°C. The bacterial solution was prepared as per the following steps: First, the feces were removed from the refrigerator at −80°C and melted in a water bath at 37°C. Next, the balance and vortex meters were placed in an anaerobic incubator, and the melted feces mixture was placed on the vortex meter in an anaerobic incubator for shaking and mixing, with each tube being shaked for 2–3 min (Zhou et al., 2017; Hu et al., 2018; Takahashi et al., 2019). A 70 μl filter mesh was used to filter the mixed feces sequentially to remove impurities from the feces. The filtered fecal microbiota fluid was centrifuged at 4000 rpm at 4°C for 15 min. The supernatant was discarded, and 160 mg/ml saline was added for resuspension. The solution was centrifuged again at 4000 rpm at 4°C for 15 min, and the supernatant was discarded. The fecal microbial protection solution was prepared as per the following steps: Normal saline containing 30% glycerol, supplemented with 0.1% L-cysteine, was subjected to 121°C sterilization for 15 min (Sun et al., 2018; Bárcena et al., 2019). Unopened protective fluid was stored at −20°C. After centrifugation, the supernatant was removed, and the remaining bacteria were directly mixed with the protective solution. A sterile 5 ml Eppendorf tube was used. The extracted fecal bacteria were divided into 4 ml per tube and stored at −80°C to avoid repeated freezing and thawing.

#### Construction of a humanized mouse model of FMT

GF mice were randomized into a control (*n* = 5) and FMT arms (*n* = 35). The control arm was gavaged with phosphate-buffered saline (PBS, PH 7.4–7.6), and the FMT arm was gavaged with human-derived fecal microbiota solution obtained during gestation twice a week at 0.2 ml/time for 3 weeks. Fresh feces were collected for 16S rDNA sequencing on day 1 before, and on days 7, 14, and 21 after FMT.

SPF mice were randomized into a control (*n* = 5) and FMT arms (*n* = 35). SPF mice were first gavaged with mixed antibiotics (ampicillin, 1 g/l; neomycin sulfate, 1 g/l; metronidazole, 1 g/l; vancomycin, 0.5 g/l) at 500 μl/mouse for 2 weeks (Schuijt et al., 2016; Wang et al., 2020; Strati et al., 2021), and the transplantation of fecal microbiota solution was started 3 days after discontinuation of antibiotic administration. Randomization and transplantation were performed in the same manner as performed in GF mice. For 16S rDNA sequencing, fresh feces were collected on day 1 before antibiotic gavage; day 1 before FMT, and days 7, 14, and 21 after FMT.

#### Extraction of genomic DNA

The genomic DNA of the samples was extracted using the Sodium Dodecyl Sulfate based method, after which the DNA purity and concentration were determined using agarose gel electrophoresis. An appropriate amount of sample DNA was placed in a centrifuge tube and diluted to 1 ng/μL using sterile water.

#### Polymerase chain reaction amplification of the V3/V4 variable regions of the gene sequence

PCR was performed using diluted genomic DNA as a template, specific barcoded primers based on the sequencing region, Phusion^®^ High-Fidelity PCR Master Mix with GC Buffer from New England Biolabs, and high-performance high-fidelity enzymes to ensure the efficiency and accuracy of the amplification.

#### Mixing and purification of PCR products

The PCR products were subjected to electrophoresis using a 2% agarose gel. The validated PCR products that were validated were purified with magnetic beads and quantified by enzyme labeling. The samples were mixed in equal amounts according to the concentration of the PCR products. After mixing the PCR products well, electrophoresis was performed on a 2% agarose gel, and the target bands were recovered using a gel extraction kit provided by Qiagen.

#### Library construction and sequencing

Library construction was performed using a TruSeq^®^ DNA PCR-Free Sample Preparation Kit, and the constructed libraries were quantified by using Qubit and quantitative PCR (Q-PCR). After library qualification, DNA sequencing was performed using a NovaSeq6000.

### Statistical processing

The effective tags of all samples were clustered using Uparse (Uparse v7.0.1001), and species annotation was performed based on an operational taxonomic unit (OTU) sequences. Species annotation was performed using Mothur with the SSUrRNA database of SILVA138.1 (threshold was set to 0.8–1). R software, Version 2.15.3 (R Core team, Vienna, Austria) was used to construct the principal coordinates analysis (PCoA) plot and the non-metric multi-dimensional scaling (NMDS) plot, and visualize the MetaStat analysis of the intergroup differences. The ggplot2 package of R software was used for PCoA analysis, and the vegan package of R software was used for the NMDS analysis. The Linear discriminant analysis Effect Size (LEfSe) analysis was performed using LEfSe software with a default screening value of 4 for the linear discriminant analysis (LDA) score. Differences were considered statistically significant at *p* < 0.05.

## Results

### Analysis of the relative abundance of gut microbial composition at the genus level

**Figure 1:**
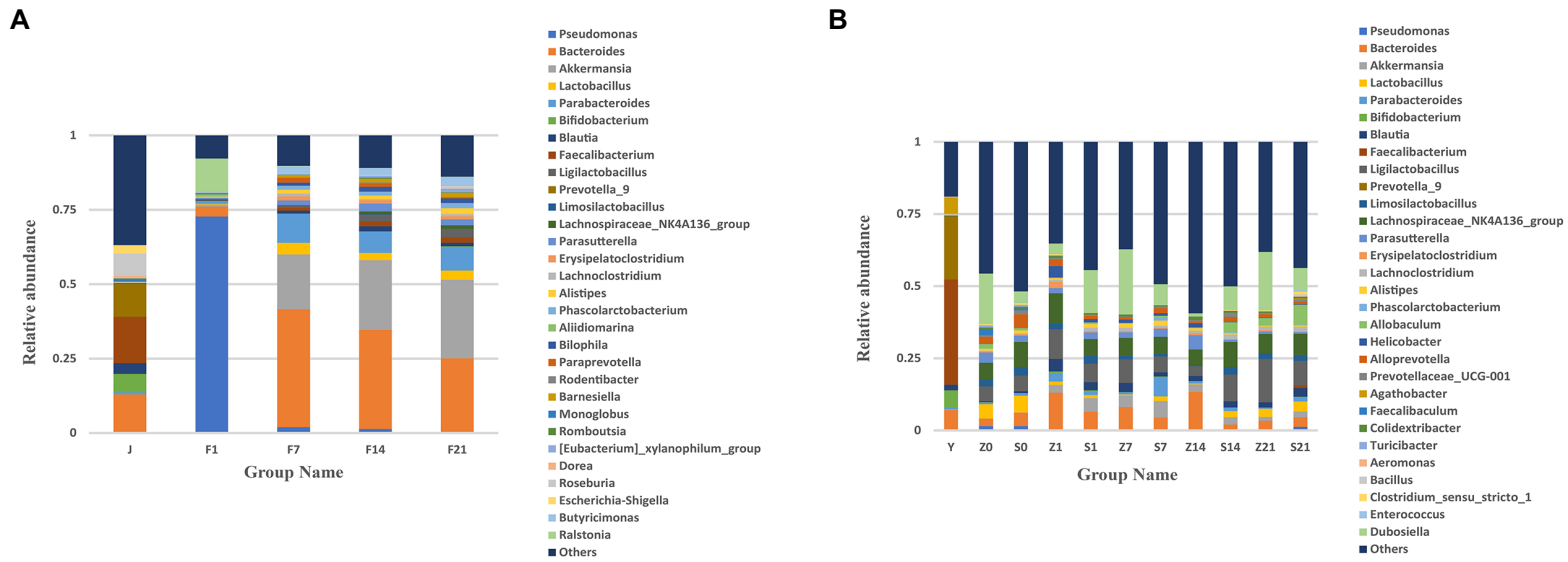
Bar chart of relative abundance of gut microbial composition at the genus level. **(A)** Bar chart of the relative abundance of gut microbial composition at the genus level of GF mice; J, human-derived mixed gestational fecal fluid; F, FMT arm; **(B)** Bar chart of the relative abundance of gut microbial composition at the genus level of SPF mice; Y, human-derived mixed gestational fecal fluid; Z, control arm; S, FMT arm; For all the pictures: 0, before gavage of antibiotics; 1, after gavage of antibiotics, 1 day before FMT; 7, day 7 of FMT; 14, day 14 of FMT; 21, day 21 of FMT. FMT, fecal microbiota transplantation; GF, germ-free; SPF, specific pathogen free.

Based on the annotation results, the top 30 genera with the maximum abundance at the genus level were selected for each arm, and a bar chart of cumulative relative abundance was produced. From the relative abundance bar chart at the genus level, the intestinal microbiota of pregnant women mainly consisted of *Bacteroides*, *Parabacteroides*, *Faecalibacterium*, *Prevotella-9*, *Roseburia*, and *Bifidobacterium*. The intestinal microbiota of the GF mice after FMT predominantly consisted of *Bacteroides*, *Akkermansia*, *Lactobacillus*, *Parabacteroides*, *Faecalibacterium*, and *Roseburia*. The composition of the microbiota on days 7 (F7), 14 (F14), and 21 (F21) was similar. However, with an increase in transplantation days, the relative abundance of genera changed across the different time points (Figure 1A). Compared with the that noted in the FMT group, the main intestinal flora did almost not change for any GF mouse, the individual differences were small, and the flora composition was similar (Supplementary Figure S2A).

The maternal gut microbiota mainly consisted of *Bacteroides*, *Faecalibacterium*, *Akkermansia*, *Blautia*, *Prevotella-9*, and *Bifidobacterium*. After FMT, the gut flora of SPF mice mainly consisted of *Dubosiella*, *Parabacteroides*, *Ligilactobacaillus*, *Bacteroides*, *Lachnospiraceae_NK4A136_group*, and *Akkermansia*. The flora composition of SPF mice did not change before and after FMT, with relative abundance of microbial flora being higher after the gavage of antibiotics (Z1 and S1) than before the gavage of antibiotics (Z0 and S0). Moreover, the relative abundance of microbial flora after FMT was not as high as that of the original microbial flora in mice (Figure 1B). From the bar chart of the relative abundance of gut microbial composition at the genus level between SPF mice in each FMT group on days 7, 14 and 21 of fecal bacteria transplantation, the main intestinal microflora were *Parabacteroides*, *Dubosiella*, *Ligilactobacaillus*, *Allobaculum*, *Akkermansia, Lachnospiraceae_NK4A136_group*, and compared to the FMT group, there was almost no change in the main intestinal flora. The microbiota composition of each mouse in the control group at each time point (S7, S14 and S21) was similar, but the microbiota of the control group on day 14 was significantly different from that on days 7 and 21. After FMT, the microbiota composition of few mice was similar, while few showed significant differences. For example: on day 7 of FMT, S6.7d-S15.7d was similar to that of the control group, S24.14d-S32.14d was similar, S33.14d-S39.14d was similar, while these factors were dissimilar in some mice. Therefore, SPF mice showed significant individual differences, and the overall composition of the microbiota was not similar (Supplementary Figure S2B).

### Table of relative abundance of gut microbial composition at the genus level

The relative abundance of 18 genera, including *Pseudomonas*, *Bacteroides*, *Ralstonia*, *Akkermansia*, *Parabacteroides*, *Lactobacillus,* and *Butyricimonas*, after FMT exceeded the relative abundance of the gestational fecal fluid (J), and the relative abundance steadily increased. However, the relative abundance of six genera, namely *Bifidobacterium, Blautia, Faecalibacterium, Prevotella_9, Roseburia* and *Romboutsia,* as well as “others” did not reach the relative abundance of the gestational fecal fluid (J); however, the bacterial abundance steadily increased with the increase in days after transplantation (Table 1). In addition to the unidentified “others,” 159 genera were detected in the bacterial liquid J. Overall, 139 genera were detected on F7 in GF mice, among which 41 had a relative abundance greater than that in J. Furthermore, 131 genera were detected on F14 in GF mice, among which 48 had a relative abundance greater than that of J. Finally, 133 genera were detected on F21 in GF mice, among which 45 were relatively more abundant than in that in J (*p* > 0.05; Supplementary File 1). Except for the unidentified “others,” 271 genera were detected before oral administration of antibiotics (S0) and 172 genera were detected after intragastric administration of antibiotics (S1). Intragastric administration of antibiotics eliminated 125 genera, of which 86 decreased in relative abundance and 60 increased. Further, additional 26 genera were detected in mice after the antibiotic administration. This shows that antibiotics have an effect, but they cannot completely eliminate all genera, and may even increase the relative abundance of some genera (Supplementary File 2). At Z7, the top 30 microbial flora relative abundance was 62.8%, while the microbial flora relative abundance of S7 was 50.7%. The relative abundance in the control group (Z7) was higher than that in the FMT group (S7). At Z14, the top 30 microbial flora relative abundance was 40.57%, while the microbial flora relative abundance of S14 was 49.97%. The relative abundance in the control group (Z14) was lower than that in the FMT group (S14). At Z21, the top 30 microbial flora relative abundance was 61.87%, while the microbial flora relative abundance of S21 was 56.29%. The relative abundance in the control group (Z21) was higher than that in the FMT group (S21). *Faecalibacterium* accounted for 36.52% of the bacterial solution and was originally not present in mice, but only colonized 0.89% on S21 after FMT (Table 2). Except for the unidentified “others,” 73 genera were detected in the bacterial solution (Y) and the SPF mice on the 7 days, of which 40 genera relative abundance was observed on S7 greater than that observed on Z7. There were 73 genera detected in the bacterial solution (Y) and the SPF mice on day 14, of which 58 had a relative abundance on S14 greater than that on Z14. There were 97 genera were detected in the bacterial solution (Y) and the SPF mice on day 21, of which 86 had a relative abundance on S21 greater than that on Z21 (*p* > 0.05; Supplementary File 3).

**Table 1:** Relative abundance of gut microbial composition at the genus level Top30 in germ free mice.

Taxonomy	J	F1	F7	F14	F21
*Pseudomonas*	0.27%	72.75%	1.98%	1.36%	0.34%
*Bacteroides*	12.70%	3.29%	39.65%	33.34%	24.73%
*Akkermansia*	0.02%	0.71%	18.39%	23.40%	26.43%
*Lactobacillus*	0.01%	0.26%	3.90%	2.41%	3.06%
*Ralstonia*	0.01%	11.16%	0.19%	0.03%	0.02%
*Parabacteroides*	0.67%	0.99%	9.82%	7.17%	8.01%
*Butyricimonas*	0.04%	0.28%	2.69%	2.33%	3.10%
*Faecalibacterium*	15.61%	0.25%	1.03%	1.60%	1.77%
*Ligilactobacillus*	0.01%	0.10%	0.81%	2.23%	2.83%
*Prevotella_9*	11.00%	0.03%	0.01%	0.09%	0.03%
*Limosilactobacillus*	0.03%	0.01%	0.01%	0.54%	0.06%
*Roseburia*	7.47%	0.03%	0.00%	0.10%	0.84%
*Lachnospiraceae_NK4A136_group*	0.27%	0.03%	0.01%	0.59%	1.14%
*Parasutterella*	0.11%	0.30%	1.60%	2.60%	2.05%
[*Eubacterium*]_xylanophilum_group	0.11%	0.00%	0.00%	0.63%	1.18%
*Bifidobacterium*	6.19%	0.04%	0.00%	0.07%	0.15%
*Erysipelatoclostridium*	0.00%	0.07%	1.38%	1.14%	1.00%
*Bilophila*	0.09%	0.19%	1.07%	1.62%	1.61%
*Blautia*	3.61%	0.19%	0.96%	1.71%	1.21%
*Paraprevotella*	0.02%	0.11%	1.59%	1.17%	0.40%
*Romboutsia*	0.49%	0.03%	0.02%	0.32%	0.09%
*Lachnoclostridium*	0.09%	0.21%	0.91%	0.36%	0.75%
*Rodentibacter*	0.00%	0.00%	0.02%	0.27%	0.01%
*Alistipes*	0.15%	0.19%	1.31%	1.15%	1.95%
*Barnesiella*	0.06%	0.10%	1.00%	1.27%	1.49%
*Phascolarctobacterium*	0.17%	0.09%	1.28%	1.37%	1.72%
*Aliidiomarina*	0.00%	0.71%	0.03%	0.00%	0.00%
*Dellaglioa*	0.00%	0.01%	0.30%	0.00%	0.00%
*Eisenbergiella*	0.00%	0.11%	0.57%	0.54%	0.49%
*Escherichia-Shigella*	2.79%	0.06%	0.03%	0.14%	0.04%
Others	38.05%	7.70%	9.45%	10.44%	13.49%

**Table 2:** Relative abundance of gut microbial composition at the genus level Top30 in specific pathogen free mice.

Taxonomy	Y	Z0	S0	Z1	S1	Z7	S7	Z14	S14	Z21	S21
*Dubosiella*	0.00%	17.42%	4.14%	3.65%	14.79%	22.58%	7.25%	1.07%	8.15%	20.33%	7.73%
*Pseudomonas*	0.00%	1.51%	1.52%	0.37%	0.04%	0.04%	0.13%	0.04%	0.00%	0.00%	1.27%
*Parabacteroides*	0.65%	0.61%	0.75%	2.91%	1.32%	0.77%	6.69%	0.79%	1.14%	0.43%	1.47%
*Ligilactobacillus*	0.00%	5.05%	5.40%	10.38%	6.32%	8.10%	5.49%	3.33%	9.26%	15.07%	8.41%
*Faecalibacterium*	36.52%	0.00%	0.00%	0.00%	0.00%	0.00%	0.00%	0.00%	0.00%	0.00%	0.89%
*Bacteroides*	7.06%	2.60%	4.65%	12.77%	6.52%	8.10%	4.30%	13.40%	2.09%	3.39%	3.34%
*Allobaculum*	0.00%	1.63%	0.78%	0.22%	0.49%	0.15%	0.40%	0.10%	3.58%	2.56%	6.95%
*Akkermansia*	0.24%	0.00%	0.07%	2.68%	4.63%	3.97%	5.83%	2.56%	2.51%	1.35%	1.97%
*Lachnospiraceae_NK 4A136_group*	0.19%	5.65%	8.78%	10.50%	5.68%	6.02%	5.94%	5.51%	8.72%	6.68%	7.30%
*Helicobacter*	0.00%	0.01%	0.11%	3.97%	1.16%	1.15%	0.91%	1.54%	0.09%	0.20%	0.19%
*Blautia*	1.91%	0.38%	0.68%	4.35%	2.84%	3.17%	1.46%	1.84%	2.13%	1.77%	3.00%
*Prevotella_9*	21.82%	0.00%	0.00%	0.00%	0.00%	0.00%	0.00%	0.00%	0.00%	0.00%	0.09%
*Lactobacillus*	0.00%	4.95%	5.79%	1.15%	1.10%	0.39%	1.56%	0.30%	2.16%	2.77%	3.46%
*Limosilactobacillus*	0.00%	2.57%	2.88%	1.86%	2.93%	1.46%	0.90%	0.28%	2.62%	1.95%	2.10%
*Alloprevotella*	0.00%	2.48%	4.69%	2.38%	1.14%	0.92%	1.79%	0.45%	1.73%	1.16%	0.93%
*Prevotellaceae*_UCG-001	0.00%	0.58%	1.38%	0.45%	0.41%	0.16%	0.50%	0.58%	1.48%	0.50%	0.83%
*Alistipes*	0.09%	0.66%	0.96%	0.60%	1.26%	1.42%	1.80%	0.85%	0.50%	0.35%	0.41%
*Erysipelatoclostridium*	0.00%	0.39%	0.50%	2.25%	0.44%	0.41%	0.34%	0.71%	0.20%	0.47%	0.23%
*Parasutterella*	0.13%	3.58%	2.15%	1.80%	2.28%	1.99%	2.78%	4.97%	0.76%	0.89%	0.59%
*Phascolarctobacterium*	0.19%	0.00%	0.00%	0.00%	0.02%	0.05%	1.22%	0.09%	0.17%	0.03%	0.21%
*Bifidobacterium*	5.98%	0.14%	0.15%	0.56%	0.31%	0.06%	0.18%	0.02%	0.06%	0.01%	0.19%
*Agathobacter*	5.67%	0.00%	0.00%	0.00%	0.00%	0.00%	0.00%	0.00%	0.00%	0.00%	0.25%
*Faecalibaculum*	0.00%	2.12%	0.60%	0.11%	0.01%	0.00%	0.00%	0.00%	0.01%	0.00%	0.00%
*Colidextribacter*	0.00%	0.51%	0.66%	0.54%	0.55%	0.72%	0.50%	1.25%	0.63%	0.46%	0.54%
*Turicibacter*	0.01%	0.80%	0.67%	0.30%	0.02%	0.00%	0.00%	0.00%	0.04%	0.05%	0.06%
*Aeromonas*	0.00%	0.00%	0.00%	0.00%	0.00%	0.00%	0.00%	0.00%	0.00%	0.00%	0.21%
*Bacillus*	0.00%	0.00%	0.00%	0.00%	0.00%	0.00%	0.00%	0.00%	0.00%	0.00%	0.56%
*Clostridium*_sensu_stricto_1	0.18%	0.44%	0.55%	0.34%	0.03%	0.00%	0.01%	0.01%	0.31%	0.24%	0.93%
*Enterococcus*	0.01%	0.00%	0.01%	0.01%	0.02%	0.02%	0.01%	0.00%	0.00%	0.00%	0.48%
*Lachnoclostridium*	0.40%	0.24%	0.34%	0.60%	1.24%	1.17%	0.70%	0.85%	1.62%	1.20%	1.69%
Others	18.94%	45.65%	51.77%	35.24%	44.44%	37.20%	49.30%	59.43%	50.03%	38.13%	43.71%

### Heat map of gut microbial composition relative abundance clustering

**Figure 2:**
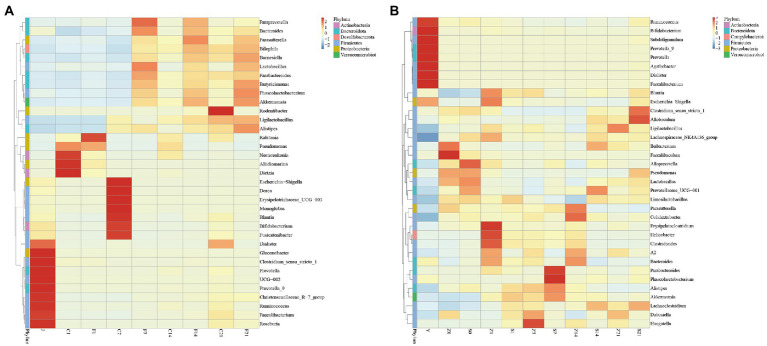
Heat map of relative abundance of gut microbial composition clustering at the genus level. **(A)** Heat map of relative abundance of gut microbial composition clustering at the genus level of GF mice; J, human-derived mixed gestational fecal fluid; C, control arm; F, FMT arm; **(B)** Heat map of relative abundance of gut microbial composition clustering at the genus level of SPF mice; Y, human-derived mixed gestational fecal fluid; Z, control arm; S, FMT arm. FMT, fecal microbiota transplantation; GF, germ-free; SPF, specific pathogen free.

Statistical analysis revealed that all genera in the bacterial fluid J were detected in the GF mice. There were 14 genera whose relative abundance on F7, F14, and F21 exceeded that of the bacterial solution (J), all of which were *Paraprevotella*, *Parabacteroides*, *Bilophila*, *Ligilactobacillus*, *Phascolarctobacterium*, *Pseudomonas*, *Butyricimonas*, *Bacteroides*, *Lactobacillus*, *Barnesiella*, *Akkermansia*, *Alistipes*, *Parasutterella*, *Ralstonia* (*p* > 0.05; Supplementary File 4). Moreover, *Bacteroides, Lactobacillus, Butyricimonas,* and *Akkermansia* were highly abundant, indicating that even bacteria with lower relative abundance in the solution multiplied rapidly in GF mice. Despite the high relative abundances of *Faecalibacterium, Bifidobacterium,* and *Blautia* in J, the relative abundance did not exceed that of the microbial solution in the GF mice (Figure 2A). After FMT, there were 12 genera with relative abundance of S7 over Z7, 12 genera with relative abundance of S14 over Z14, and 20 genera with relative abundance of S21 over Z21 (*p* > 0.05; Figure 2B; Supplementary File 5).

### Analysis of the level of difference in microbial flora before and after FMT

**Figure 3:**
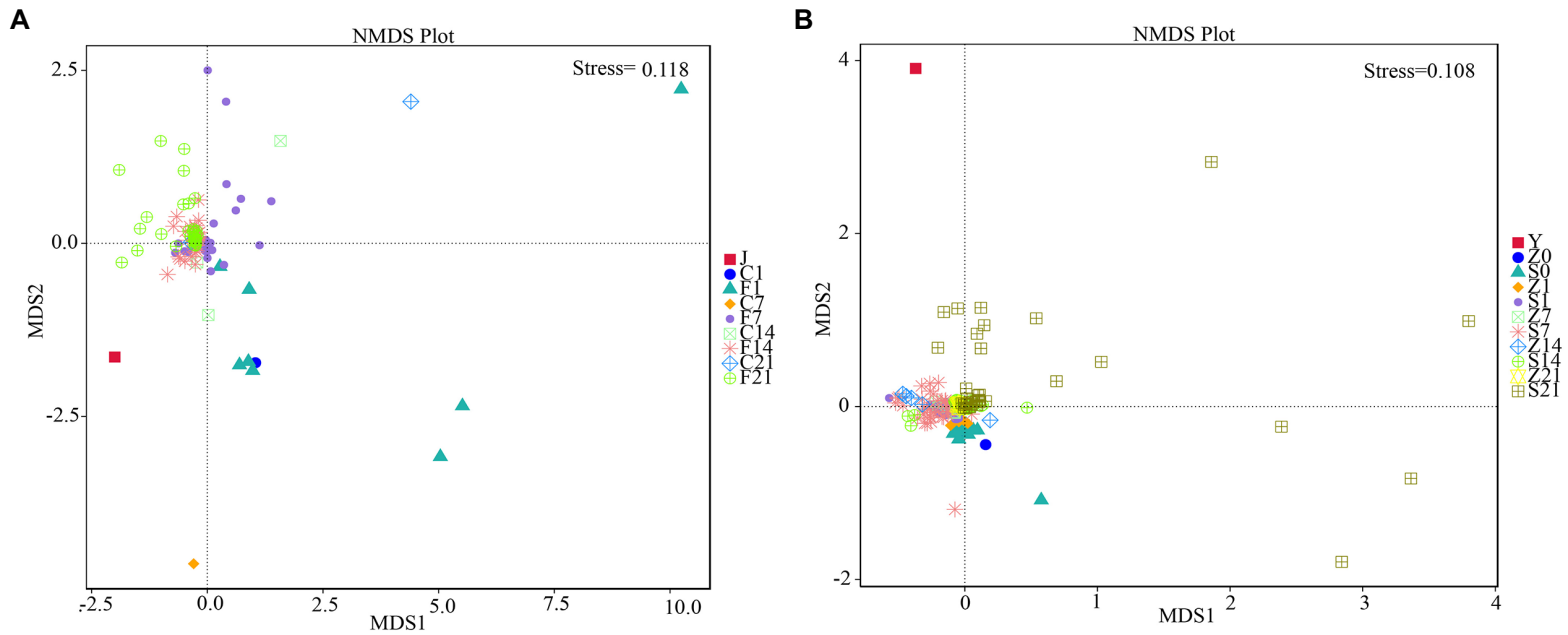
Non-metric multi-dimensional scaling analysis. **(A)** NMDS analysis in GF mice; **(B)** NMDS analysis in SPF mice. GF, germ-free; NMDS, non-metric multi-dimensional scaling; SPF, specific pathogen free.

Non-Metric Multi-Dimensional Scaling (NMDS) is a nonlinear model based on the Bray-Curtis distance. The species information contained in the sample is reflected on a two-dimensional plane in the form of points. The degree of difference between different samples is reflected by the distance between points, which can reflect the inter-group and intra-group differences of the samples. A stress of less than 0.2 indicated that the NMDS could accurately reflect the degree of variation between samples. The NMDS analysis results for GF mice showed that the microbial flora of each control group clustered together before FMT, and the flora on days 7 (F7), 14 (F14), and 21 (F21) after FMT clustered together and were closer to the gestational flora (J) (Figure 3A). The NMDS analysis results for SPF mice showed that only day 21 after FMT (S21) was scattered away from the control group, while fecal bacteria were clustered with the control arm on both days 7 (S7) and 14 (S14) after FMT, with both being distant from the gestational flora (Y) (Figure 3B). The flora of GF mice differed greatly before and after FMT, and the flora after FMT showed little difference from the gestational flora, indicating significant effects. However, for SPF mice, a good transplantation outcome was only observed on S21, and the flora were substantially different from the gestational flora.

### Similarity analysis of the microbial flora before and after FMT

**Figure 4:**
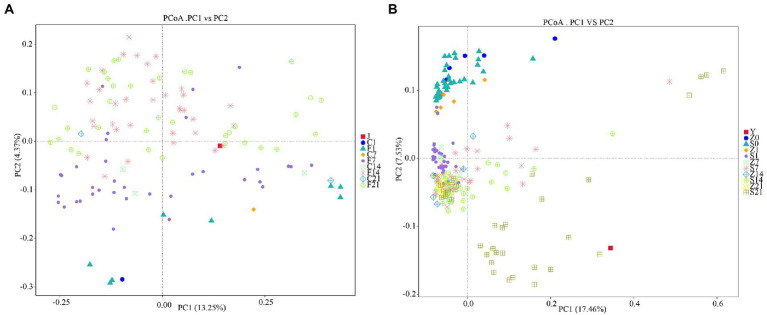
Principal coordinates analysis based on unweighted_unifrac distance. **(A)** PCoA analysis in GF mice; **(B)** PCoA analysis in SPF mice. GF, germ-free; PCoA, principal coordinates analysis; SPF, specific pathogen free.

Principal coordinate analysis (PCoA) involves the extraction of the most dominant elements and structures from multi-dimensional data by ranking of a series of eigenvalues and eigenvectors. The closer the samples are, the more similar the structural composition of the gut microbes. Therefore, samples with high similarity in community structure tended to cluster together, whereas samples with highly different communities tended to be far apart. The PCoA plots showed that the colonies in the control arms before FMT in GF mice clustered together, and that the microbial colonies on days 7 (F7), 14 (F14), and 21 (F21) after FMT clustered together with the gestation fluid (J) and were very similar to each other (Figure 4A). PCoA plots of SPF mice showed that most colonies before (Z0, S0) and after (Z1, S1) gavage with antibiotics were some certain distance away, whereas a small fraction remained indistinguishable. Only colonies from S21 were distant from the control arm and were similar to the gestational microbial solution (Y), whereas the rest of the groups were clustered together and were different form the microbial solution (Figure 4B). In GF mice, the colonies differed greatly before and after FMT, and were similar to the fecal microbial solution during gestation, demonstrating a significant effect. However, only S21 demonstrated a significant effect in SPF mice, and the microbiota before and after antibiotic administration could not be clearly distinguished.

### Metastat analysis of the intergroup differences

**Figure 5:**
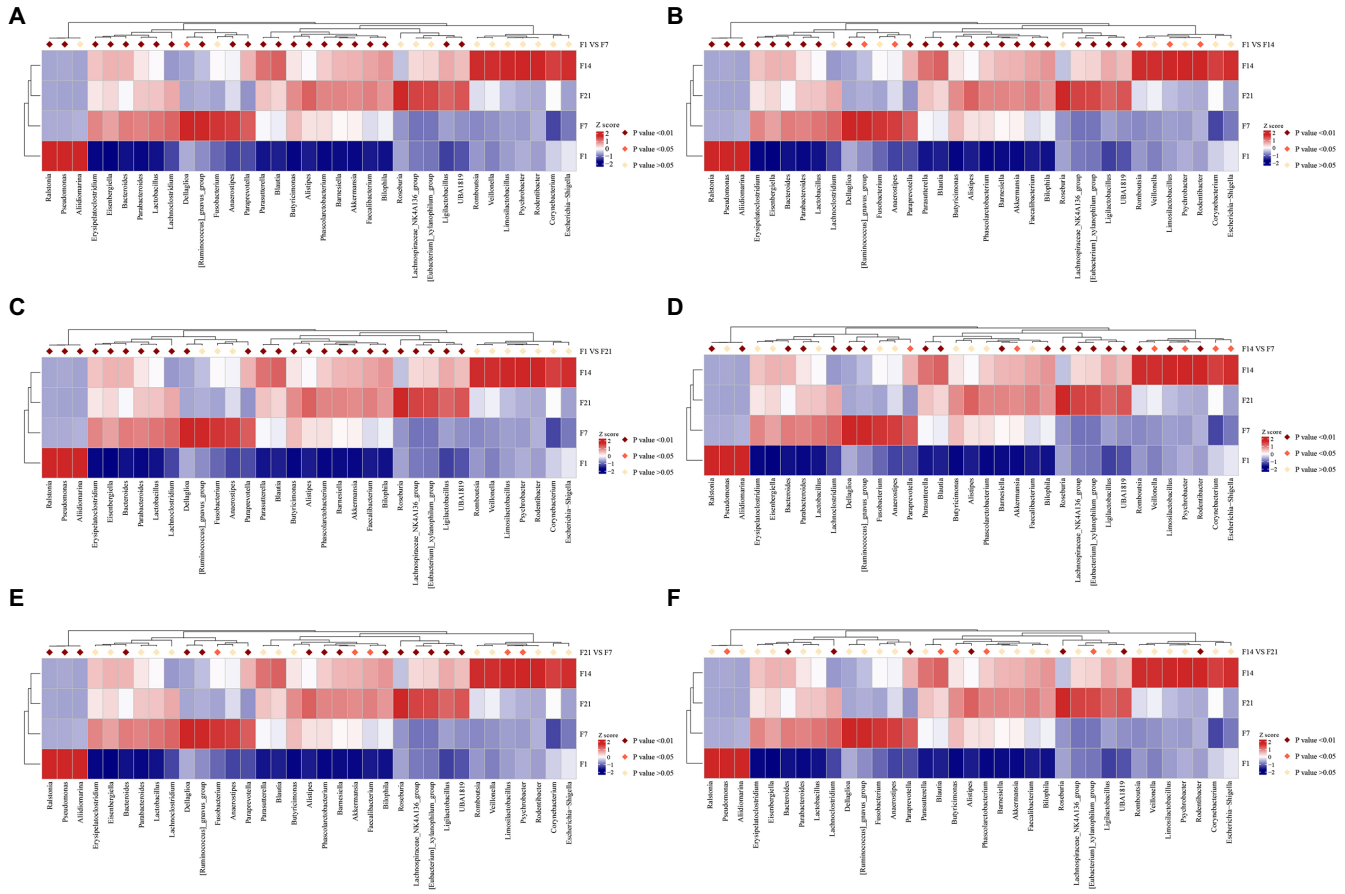
F1, day 1 of FMT; F7, day 7 of FMT; F14, day 14 of FMT; F21, day 21 of FMT; **(A)** F1 VS F7, Analysis of the intergroup differences on the day 1 and 7 of fecal microbiota transplantation; **(B)** F1 VS F14, Analysis of the intergroup differences on the day 1 and 14 of fecal microbiota transplantation; **(C)** F1 VS F21, Analysis of the intergroup differences on the day 1 and 21 of fecal microbiota transplantation; **(D)** F7 VS F14, Analysis of the intergroup differences on the day 7 and 14 of fecal microbiota transplantation; **(E)** F7 VS F21, Analysis of the intergroup differences on the day 7 and 21 of fecal microbiota transplantation; **(F)** F14 VS F21, Analysis of the intergroup differences on the day 14 and 21 of fecal microbiota transplantation. MetaStat analysis of the intergroup differences at the genus level of GF mice. *p **≤** 0.05, **p **≤** 0.01. GF, germ-free.

**Figure 6:**
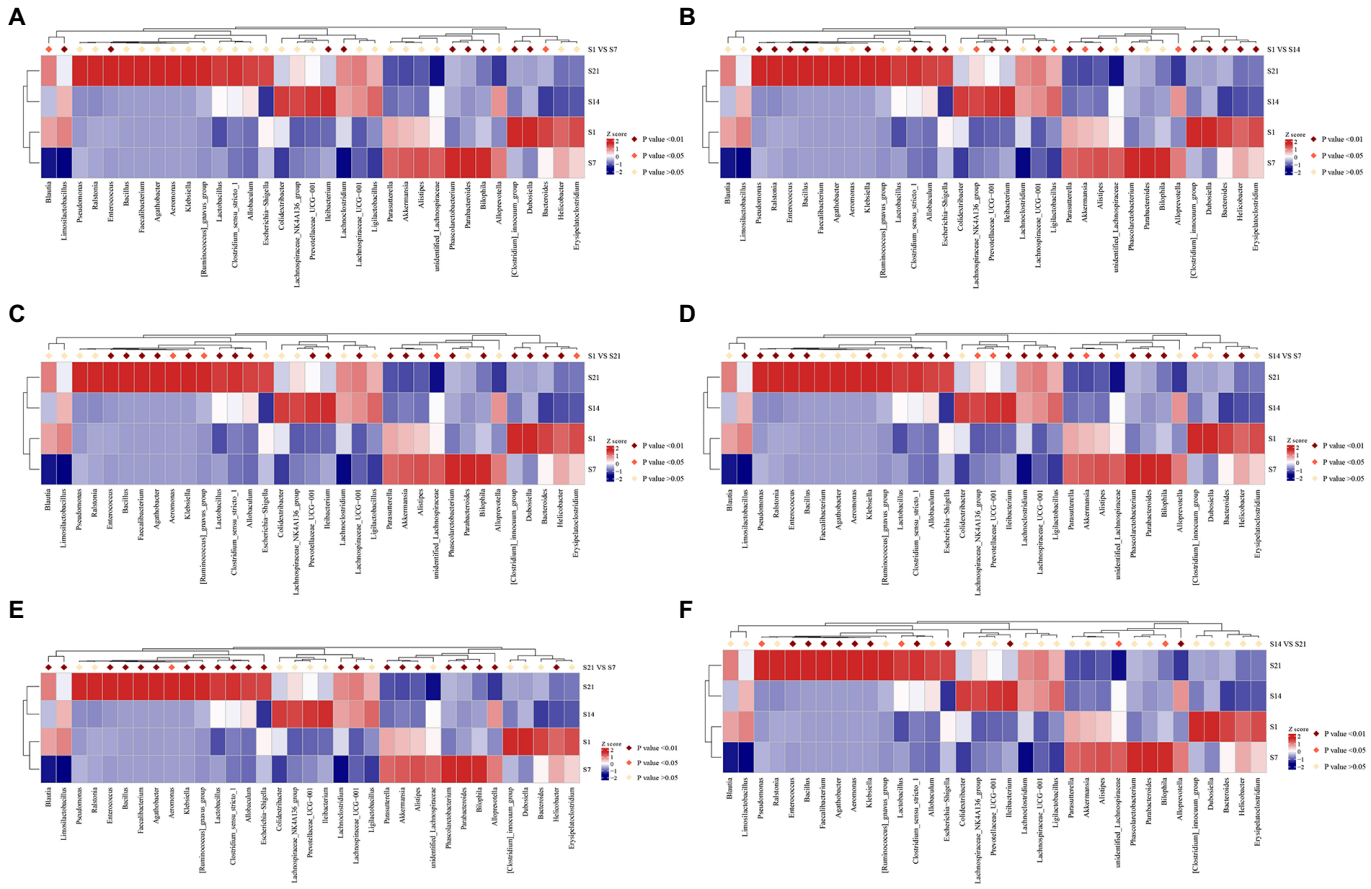
S1, day 1 of FMT; S7, day 7 of FMT; S14, day 14 of FMT; S21, day 21 of FMT; **(A)** S1 VS S7, Analysis of the intergroup differences on the day 1 and 7 of fecal microbiota transplantation; **(B)** S1 VS S14, Analysis of the intergroup differences on the day 1 and 14 of fecal microbiota transplantation; **(C)** S1 VS S21, Analysis of the intergroup differences on the day 1 and 21 of fecal microbiota transplantation; **(D)** S7 VS S14, Analysis of the intergroup differences on the day 7 and 14 of fecal microbiota transplantation; **(E)** S7 VS S21, Analysis of the intergroup differences on the day 7 and 21 of fecal microbiota transplantation; **(F)** S14 VS S21, Analysis of the intergroup differences on the day 14 and 21 of fecal microbiota transplantation. MetaStat analysis of the intergroup differences at the genus level of SPF mice. *p **≤** 0.05, **p **≤** 0.01. SPF, specific pathogen free.

Annotation heatmap plots of intergroup differences at the genus level in the GF mice revealed 23 significantly different genera between F1 and F7. Furthermore, there were 28 significantly different genera between F1 and F14 and 25 significantly different genera between F1 and F21 (*p* < 0.01; Figures 5A–C). Annotation heatmap plots of intergroup differences at the genus level in SPF mice revealed 11 significantly different genera between S1 and S7. Four genera increased in abundance after FMT. Furthermore, there were 23 significantly different genera between S1 and S14, of which nine showed increased abundance after FMT. There were 24 significantly different genera between S1 and S21, of which 14 increased in abundance after FMT (Figures 6A–C). With the increase in transplantation days, the number of significantly different genera gradually increased, indicating that the flora were gradually transplanted into the mice. However, the abundance of almost half of these genera in SPF mice after FMT was lower than that in the control group. Moreover, the growth of the original microbial flora in mice and that of humanized gestational flora could not be distinguished. A total of 25 significantly different genera were observed between F7 and F14 in the GF mice, while 21 significantly different genera were observed between F7 and F21 in the GF mice, and 12 significantly different genera were found between F14 and F21 in the GF mice (Figures 5D–F). A total of 24 significantly different genera were observed between S7 and S14 in the SPF mice. A total of 23 significantly different genera were observed between S7 and S21 in the SPF mice. There were 14 significantly different genera between S14 and S21 in the SPF mice (Figures 6D–F). With an increase in transplantation days, the number of significantly different genera gradually decreased, indicating that bacterial colonization in the mice tended to be stable.

### Analysis of intergroup differences in community structure

**Figure 7:**
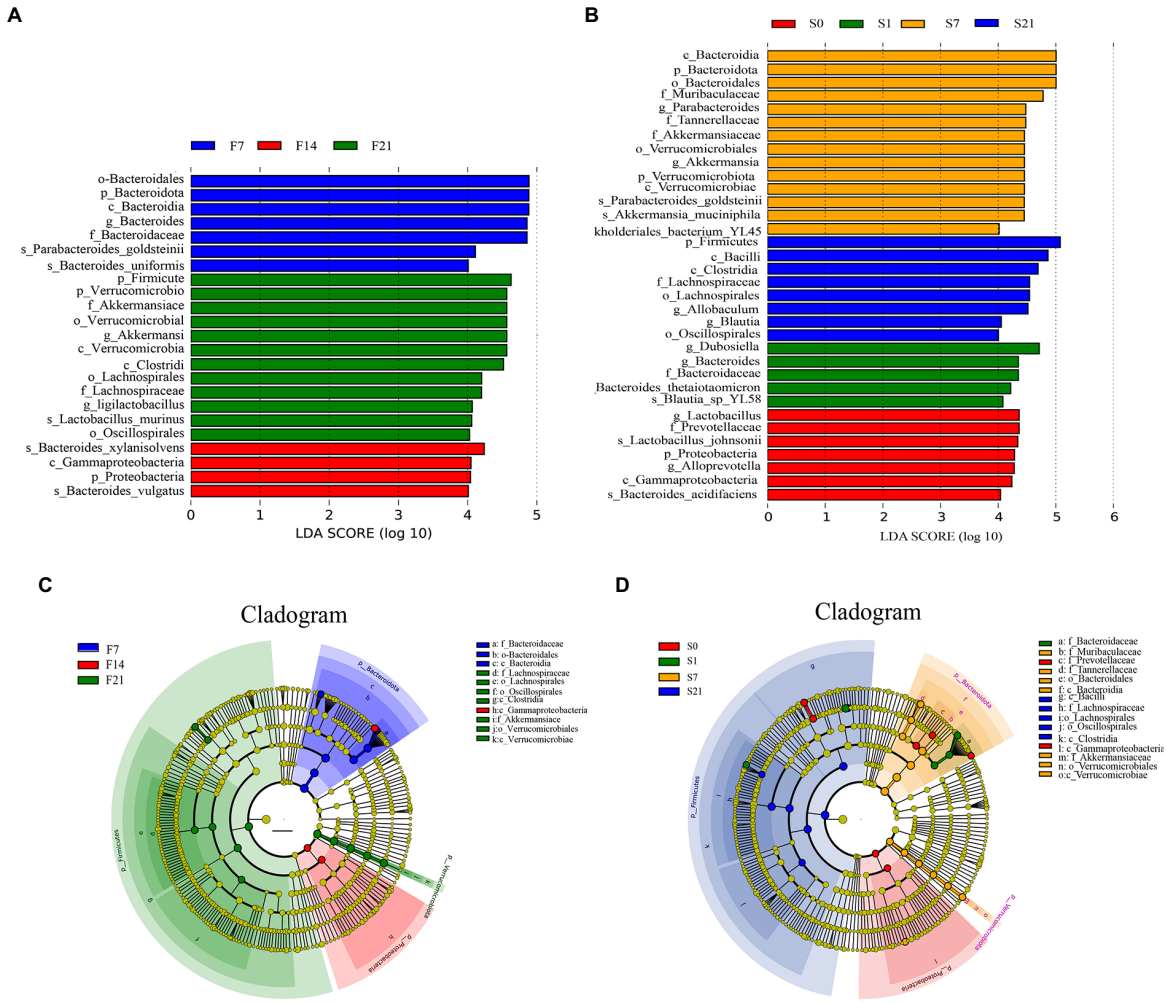
Linear discriminant analysis Effect Size analysis of intergroup differences in community structure. **(A)** Bar chart of LDA score distribution in GF mice; **(B)** Bar chart of LDA score distribution in SPF mice; **(C)** Cladogram of GF mice; **(D)** Cladogram of SPF mice. GF, germ-free; LDA, linear discriminant analysis; LefSe, Linear discriminant analysis Effect Size; SPF, specific pathogen free.

Differential analysis by LefSe showed that the order *Bacteroidales*, phylum *Bacteroidota*, and class *Bacteroidia* and so on were all significantly enriched on day 7 after the FMT, while *Bacteroides xylanisolvens*, class *Gammaproteobacteria*, phylum *Proteobacteria*, and *Bacteroides vulgatus* were significantly enriched on day 14 of fecal microbial transplantation, and the phylum *Firmicutes*, family *Akkermansiacc*, and genus *Ligilactobacillus* and so on were significantly enriched on day 21 after the FMT in the GF mice (Figures 7A,C).

Differential analysis by LefSe showed that the genus *Lactobacillus* and family *Prevotellaceae* and so on were significantly enriched on day 0; the genus *Dubosiella* and *Bacteroides* and so on were significantly enriched on day 1 before the FMT; the class *Bacteroidia* and genus *Parabacteroides* and so on were significantly enriched on day 7 after the FMT, while the phylum *Firmicutes* and class *Bacilli* and so on were significantly enriched on day 21 after the FMT in the SPF mice (Figures 7B,D).

## Discussion

No abnormalities were observed in the GF mice during or after the FMT of human-derived gestational feces. The major gut microbiota during gestation was transplanted into GF mice, and had increased gradually and steadily with the number of days of FMT. Along with two common probiotics, namely *Lactobacillus* and *Bifidobacterium*, which colonized in GF mice, *Akkermansia, Blautia,* and *Faecalibacterium* were also found. *Akkermansia* is a promising potential probiotic. Zhang et al. and Yu et al. found that *Akkermansia* improved inflammatory bowel disease and hyperlipidemia (Zhang et al., 2020; Yu et al., 2021), thus improving overall human health. *Blautia* is also considered beneficial for bacteria improving intestinal health and preventing neutropenic fever (Rashidi et al., 2022). *Faecalibacterium* can potentially improve nonalcoholic fatty liver disease (Iino et al., 2019). The relative abundance of *Bifidobacterium* in the top 30 genera of human fecal bacteria was 6%, but the colonization rate in both SPF and GF mice was only 0.06–0.19%. The relative abundance of genus *Prevotella 9* was 11–21.82%, but the colonization rate in both SPF and GF mice was only 0.01–0.09%. According to a previous study, *Bifidobacterium* can regulate the intestinal flora, prevent *Clostridioides difficile* infection, and improve intestinal health (Yang and Yang, 2021). *Prevotella 9* exerts a pro-inflammatory effect and may cause infection (Tett et al., 2021). The relative abundance of these two genera is relatively low in mice, which may be due to species differences between the donors and recipients as well as individual differences. Moreover, the dietary habits and living environments of humans and mice differ, indicating that it is impossible for the two to have identical gut microbiota. The existence of specific microbes in humans and mice may affect the construction of humanized mouse models, particularly if these microbes have host-specific physiological effects, which would result in FMT allowing only a certain fraction of the gut microbiota to colonize within the mice (Chung et al., 2012).

Since GF mice were used, there should not have been any bacteria on the day before FMT. However, bacteria were found, which were suspected to have originated from the feed. The feed was subjected to 16S rDNA testing, and many types bacteria were found in the feed. *Pseudomonas* accounted for the most bacteria on F1 and was also present in the feed. Moreover, genera, such as *Bacteroides* and *Blautia* were identical to those present in the feed before FMT. Therefore, most of the flora present before FMT were determined to be from the feed. The feed had been sterilized by irradiation, and the dead bacteria that were present would not have colonized the mice and would have been excreted by defecation. The samples were possibly contaminated while they were being moved and tested, causing the detection of bacteria in GF mice (Supplementary Figure S3). The composition of the main flora of J and Y were similar; however, some genera and relative abundances were different. This may be because the intragastric administration of fecal bacterial fluid and time of detection are inconsistent, resulting in varied compositions and abundances of detected bacterial flora and deviation of the results. However, fecal liquid is prepared at the same time and treated following the same freezing/thawing protocol. The number of bacteria transferred was the same between groups during the experiment. Therefore, this should have had little effect on the results.

SPF mice were gavaged with antibiotics for 14 days, stopped for 3 days, and then subjected to a three-week FMT. No abnormalities were observed in the SPF mice during or after the FMT of human-derived gestational feces. After antibiotic gavage, an increase in the overall microbial flora abundance in SPF mice was observed, particularly in *Parabacteroides, Bacteroides, Ligilactobacillus, Akkermansia,* and *Helicobacter*, indicating that antibiotics had no significant effect on the clearance of endogenous microorganisms in SPF mice. Several studies have attempted to deplete the mouse gut microbiota with a mixture of antibiotics of different species, doses, and treatment durations (Wiesner et al., 2001; Gopalakrishnan et al., 2021; Zhang et al., 2021) that broadly target Gram-positive, Gram-negative, and anaerobic bacteria and can reduce bacteria in the respiratory tract and mouse vagina. However, these regimens may not affect the bacterial community on the skin surface (Kennedy et al., 2018). Moreover, a study by Lundberg et al. also showed that antibiotics could not completely eradicate bacteria in mice (Lundberg et al., 2016). The gut microbiota of humans and mice were 90 and 89% similar at the phylum and genus levels, respectively, (Krych et al., 2013), indicating a high degree of similarity in the gut microbiota of humans and mice. However, closer inspection revealed key differences in microbial characteristics, particularly in microbial composition and abundance (Park and Im, 2020). For example, *Bacteroides* and *Akkermansia* are common in pregnant women and mice, but differ in abundance, whereas *Ligilactobacillus* is unique to mice. Li et al. suggested that the colonization success rate tended to be higher for homozygous strains than for new species (Li et al., 2016). After FMT, the overall number of microorganisms in SPF mice did not increase compared to that in the pre-FMT and control mice. The flora on S7, S14, and S21 were complex and changed over time, and no stable growth was observed. Moreover, the growth of the original microbial flora in the mice or that of the humanized gestational flora could not be distinguished. Observations on S7 and S14 were not distinguishable from that in the controls and were not similar to that in Y. Observations on S21 were similar to those on Y, which showed that day 21 was the better time for human-derived FMT.

Colonization of the intestine in recipient mice after FMT is fundamental to the effectiveness of FMT. Moreover, the differences in species and genera of bacteria between the donor and host as well as donor selection are critical to the success of FMT (Li et al., 2021). The colonization of the recipient by donor fecal bacteria is influenced, in part, by the gut microbial composition of the recipient (Groot et al., 2017). The GF mice did not contain any microbial flora, which greatly reduced the difficulty of FMT, and therefore better results were obtained in GF mice in this study. In contrast, SPF mice had inherent gut microbiota, which could not be eliminated even by antibiotics and added to the difficulty of FMT. The best outcome was observed on day 21 of FMT in both GF and SPF mice, which was inconsistent with the findings of Roy et al. (2019) that showed favorable results in GF mice only until week 9 after FMT. The results of this study showed that the microbial flora was highly similar to the microbial solution at 21 days, This difference in results may be due to the different types of feces transplanted and the corresponding periodicity. Therefore, many issues remain to be resolved in the construction of a humanized mouse model of FMT. Furthermore, a uniform standard for the FMT method and the criteria for successful transplantation have not yet been established. Further research is warranted to determine whether the microbial flora would be stable in mice over a longer intervention cycle.

## Conclusion

The experimental results showed that GF mice might be a better choice for the construction of a humanized FMT model of feces during pregnancy than SPF mice, A decrease in significantly different genera was noted on day 14 of FMT in both SPF mice and GF mice, indicating no stable colonization of fecal bacteria on day 14 of FMT. The best results were obtained on day 21 of FMT, in which the microbial flora were the most similar to the human-derived microbial solution obtained during pregnancy, making it the better experimental time for the humanized mouse model of gestational FMT. The human-derived gestational fecal microbiota solution was transplanted into GF and SPF mice to mimic the human gut microecology during pregnancy and construct a humanized mouse model of FMT to provide a good animal model for the study of nutritional health and disease during pregnancy, which is the basis for clinical experiments and thus provides key support for clinical experiments during pregnancy.

## Data availability statement

The raw sequence data reported in this paper have been deposited in the Genome Sequence Archive in National Genomics Data Center,China National Center for Bioinformation / Beijing Institute of Genomics, Chinese Academy of Sciences(GSA: CRA008509) that are publicly accessible at https://ngdc.cncb.ac.cn/gsa.

## Ethics statement

The animal study was reviewed and approved by the laboratory animal use and management committee of Jiangsu GemPharmatech Co., Ltd. (welfare ethics No.: GPTAP20220321-1). The laboratory animal use and management committee of Sino Animal (Beijing) Science and Technology Development Co., Ltd. (ethics No. 20210118YZE-3R).

## Author contributions

YW, ZZ and YW the experimental protocol was designed. YW conducted the experiments and wrote the first draft of the manuscript. YW and BL performed the statistical analysis of the data, and all authors participated in the paper and approved the submitted version.

## Funding

This work was funded by the National Natural Science Foundation of China (grant no. 32072191), Beijing Science and Technology Plan (grant no. Z201100002620005), and Beijing Innovation Team of Livestock Industry Technology System.

## Conflict of interest

YW, ZZ, BL, JZ, XL, and LC were employed by Beijing Sanyuan Foods Co., Ltd.

The remaining authors declare that the research was conducted in the absence of any commercial or financial relationships that could be construed as a potential conflict of interest.

## Publisher’s note

All claims expressed in this article are solely those of the authors and do not necessarily represent those of their affiliated organizations, or those of the publisher, the editors and the reviewers. Any product that may be evaluated in this article, or claim that may be made by its manufacturer, is not guaranteed or endorsed by the publisher.

## Abbreviations

FMT: Fecal microbiota transplantation
GF: Germ-free
SPF: specific pathogen free
PBS: phosphate-buffered saline
OTU: operational taxonomic unit
PCR: polymerase chain reaction
Q-PCR: quantitative polymerase chain reaction
PCoA: principal coordinates analysis
NMDS: non-metric multi-dimensional scaling
LefSe: Linear discriminant analysis Effect Size
LDA: linear discriminant analysis
F1: day 1 before FMT
F7: day 7 after FMT
F14: day 14 after FMT
F21: day 21 after FMT
S1: day 1 before FMT
S7: day 7 after FMT
S14: day 14 after FMT
S21: day 21 after FMT
Z0: control arm before gavage of antibiotics
Z1: control arm day 1 before FMT
S0: FMT arm before gavage of antibiotics
