# Supplementary material for: A study on the method and effect of the construction of a humanized mouse model of fecal microbiota transplantation
Source: Front Microbiol. 2022 Nov 16;13:1031758. doi: 10.3389/fmicb.2022.1031758 (PMC9709132; doi:10.3389/fmicb.2022.1031758)
Supplement: Supplementary file 1 [file Data_Sheet_2.ZIP › Supplementary materials/Supplementary materials Captions.docx]

Supplementary Figure 1 Bar chart of relative abundance of gut microbial composition at the genus level of 15 days of mixed feces from nine pregnant volunteers

A1-A9, nine volunteers.

Supplementary Figure 2 Bar chart of relative abundance of gut microbial composition at the genus level of each mouse

(A) Bar chart of the relative abundance of gut microbial composition at the genus level of each GF mouse

S189F7-S81F7 (7 days), S189F14-S81F14(14 days), S189F21-S81F21 (21 days)

(B) Bar chart of the relative abundance of gut microbial composition at the genus level of each SPF mouse

Control group：S1.7d- S5.7d (7 days)，S1.14d- S5.14d (14 days)，S1.21d -S5.21d (21 days)

FMT group：S6.7d- S40.7d (7 days), S6.14d- S40.14d (14 days), S6.21d -S40.21d (21 days)

FMT, fecal microbiota transplantation; GF, germ-free; SPF, specific pathogen free

Supplementary Figure 3 Bar chart of relative abundance of at the genus level in GF mouse feed

L1, first feed batch; L2, second feed batch.

GF, germ-free

Supplementary File 1. Table of the relative abundance of all intestinal microbial compositions detected at the genus level in germ-free (GF) mice

J, human-derived mixed gestational fecal fluid; F7, day 7 after FMT; F14, day 14 after FMT; F21, day 21 after FMT

Supplementary File 2. Table of the relative abundance of all intestinal microbiota compositions detected at the genus level before and after antibiotics treatment in specific pathogen free (SPF) mice

S0, before gavage of antibiotics; S1, day 1 before FMT, after gavage of antibiotics

Supplementary File 3. Table of the relative abundance of all intestinal microbial compositions detected at the genus level in specific pathogen free (SPF) mice

S7, day 7 after FMT; S14, day 14 after FMT; S21, day 21 after FMT

Z7, day 7 control arm; Z14, day 14 control arm; Z21, day 21 control arm

Supplementary File 4. The corresponding table for the heat map of relative abundance of gut microbial composition clustering at the genus level in the GF mice

J, human-derived mixed gestational fecal fluid; F7, day 7 after FMT; F14, day 14 after FMT; F21, day 21 after FMT

Supplementary File 5. The corresponding table for the heat map of relative abundance of gut microbial composition clustering at the genus level in the SPF mice

S7, day 7 after FMT; S14, day 14 after FMT; S21, day 21 after FMT

Z7, day 7 control arm; Z14, day 14 control arm; Z21, day 21 control arm
