# Supplementary figures and images for: A study on the method and effect of the construction of a humanized mouse model of fecal microbiota transplantation
Source: Front Microbiol. 2022 Nov 16;13:1031758. doi: 10.3389/fmicb.2022.1031758 (PMC9709132; doi:10.3389/fmicb.2022.1031758)

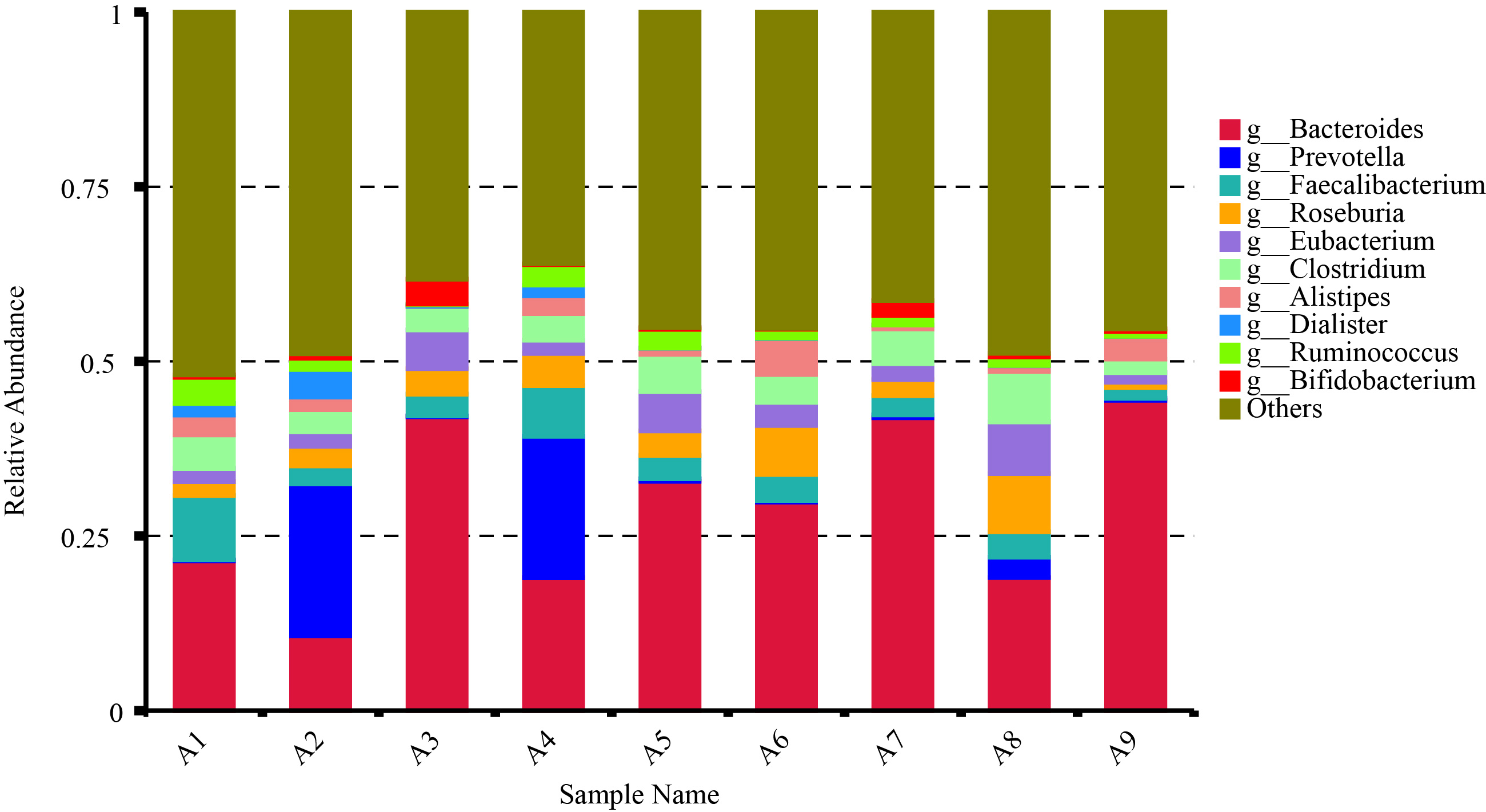

Supplement: Supplementary file 1 [file Data_Sheet_2.ZIP › Supplementary materials/Supplementary Figure 1.jpg]

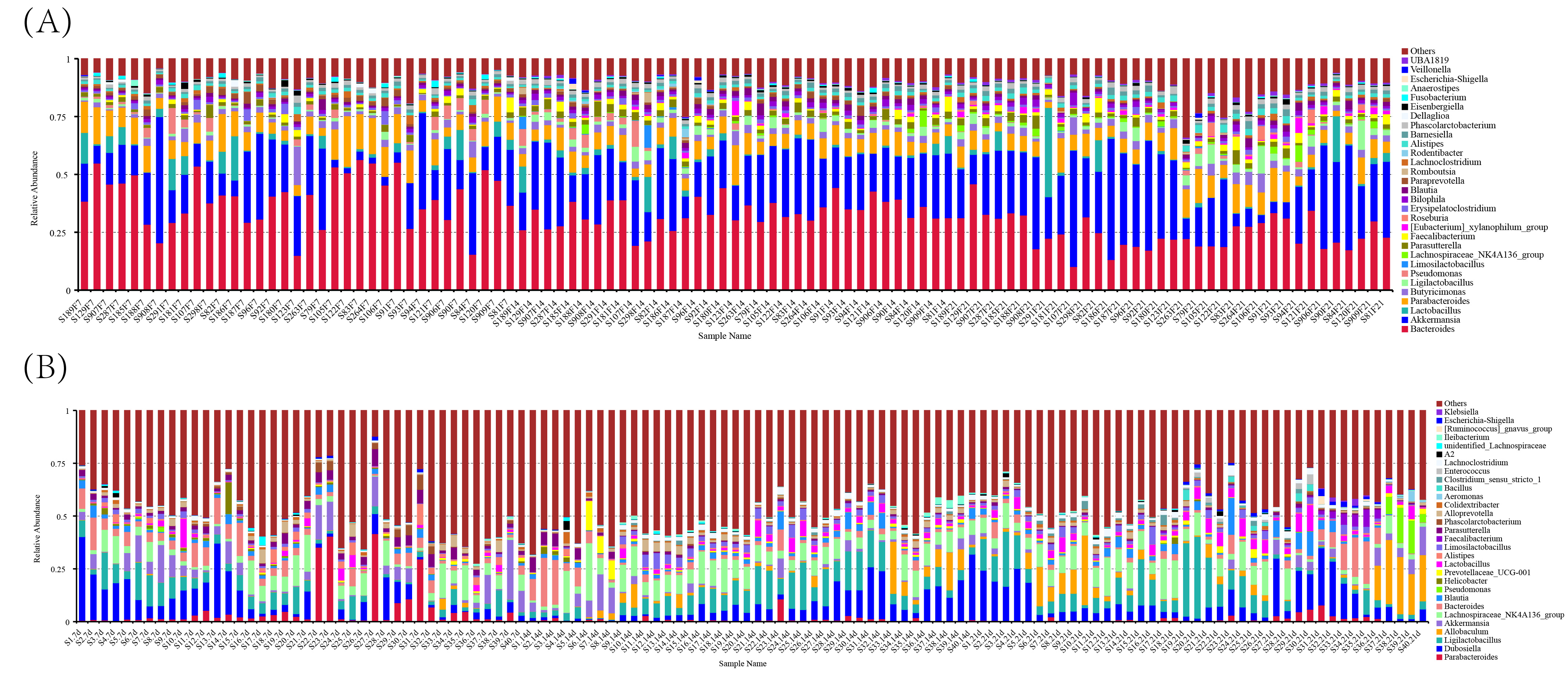

Supplement: Supplementary file 1 [file Data_Sheet_2.ZIP › Supplementary materials/Supplementary Figure 2.jpg]

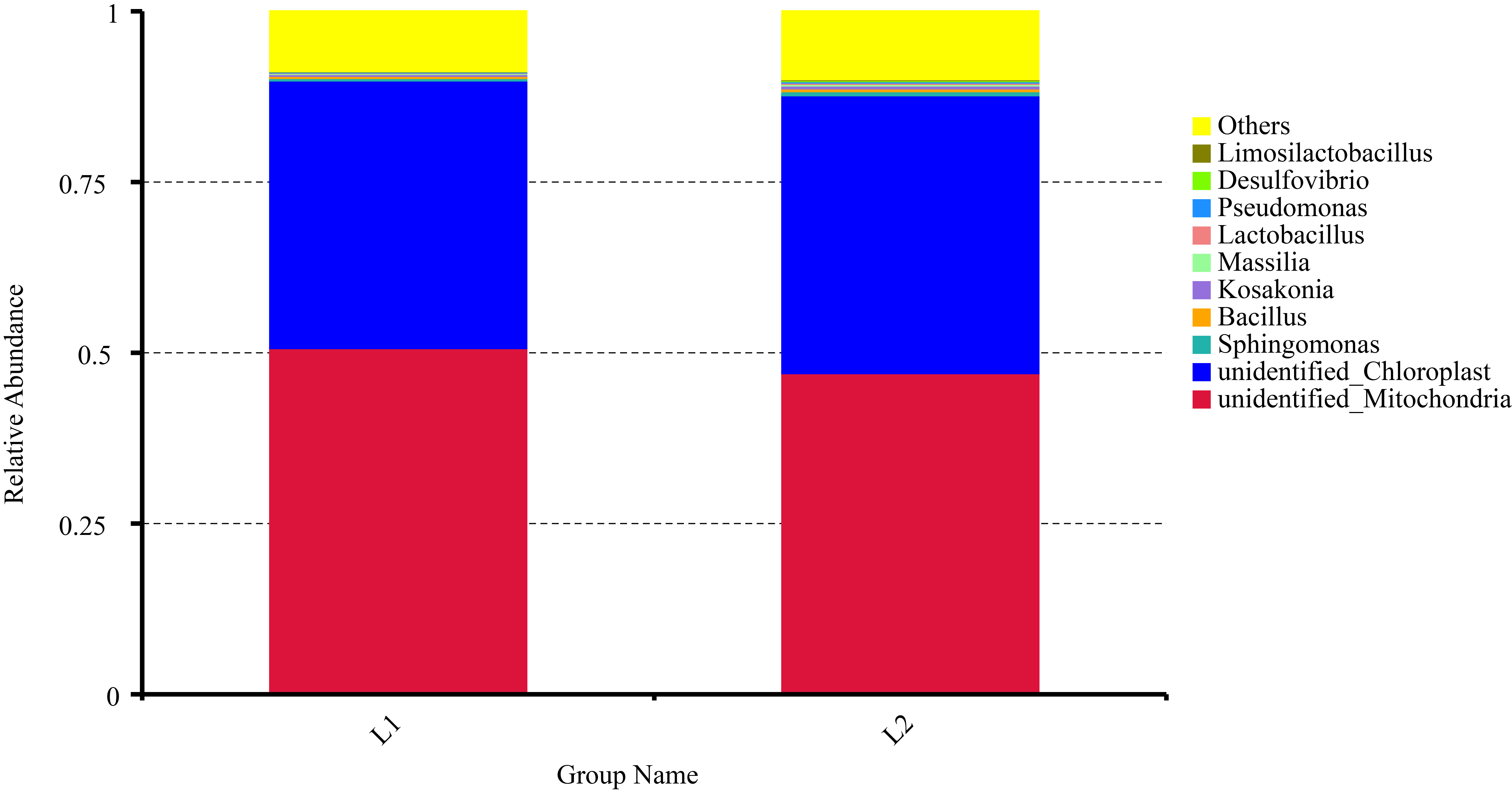

Supplement: Supplementary file 1 [file Data_Sheet_2.ZIP › Supplementary materials/Supplementary Figure 3.jpg]
